# Association between tongue pressure and subclinical carotid atherosclerosis in relation to platelet levels in hypertensive elderly men: a cross-sectional study

**DOI:** 10.1186/s12199-018-0720-5

**Published:** 2018-07-18

**Authors:** Yuji Shimizu, Shimpei Sato, Yuko Noguchi, Jun Koyamatsu, Hirotomo Yamanashi, Miho Higashi, Mako Nagayoshi, Shin-Ya Kawashiri, Yasuhiro Nagata, Noboru Takamura, Takahiro Maeda

**Affiliations:** 1 Department of Community Medicine, Nagasaki University Graduate School of Biomedical Sciences, Nagasaki, Japan; 2 Department of Cardiovascular Disease Prevention, Osaka Center for Cancer and Cardiovascular Disease Prevention, Osaka, Japan; 3 Department of Island and Community Medicine, Nagasaki University Graduate School of Biomedical Sciences, Nagasaki, Japan; 4 Department of Global Health, Medicine and Welfare, Nagasaki University Graduate School of Biomedical Sciences, Nagasaki, Japan; 5 Center for Comprehensive Community Care Education, Nagasaki University Graduate School of Biomedical Sciences, Nagasaki, Japan

**Keywords:** Atherosclerosis, Hypertension, Platelets, Tongue pressure

## Abstract

**Background:**

Age-related low-grade inflammation causing endothelial disruption influences sarcopenia, hypertension, and atherosclerosis. We reported previously that maintenance of muscle strength in elderly hypertensive men with high platelet levels is positively associated with subclinical atherosclerosis but not in those with low platelet levels. Since reduced tongue pressure is related to sarcopenia, tongue pressure may be associated with subclinical carotid atherosclerosis in hypertensive elderly subjects, and platelet levels may function as an indicator of the association between tongue pressure and subclinical carotid atherosclerosis.

**Methods:**

We conducted a cross-sectional study of 342 hypertensive elderly Japanese men aged 60–89 who participated in an annual health check-up in 2015 and 2016. Subclinical carotid atherosclerosis was defined as a common carotid intima-media thickness (CIMT) of 1.1 mm or more.

**Results:**

In the overall study population, 171 subjects demonstrated low platelets (< 21.4 × 10^4^/μL). Tongue pressure was significantly inversely associated with subclinical carotid atherosclerosis in these subjects, but not in subjects with high platelets. The known cardiovascular risk factor adjusted odds ratios (ORs) and 95% confidence intervals (CIs) of subclinical carotid atherosclerosis for a 1 standard deviation (SD) increment in tongue pressure (10.4 kPa) were 0.54 (0.35, 0.85) and 1.31 (0.87, 1.96), respectively.

**Conclusion:**

Tongue pressure is inversely associated with subclinical carotid atherosclerosis in hypertensive elderly men with low platelet levels, but not in those with high levels. This finding may thus constitute an efficient tool for clarifying the background mechanism of age-related diseases such as sarcopenia, hypertension, and atherosclerosis.

## Background

Age-related inflammation, namely chronic low-grade inflammation, has been revealed to disrupt the microvascular endothelium and thus impair blood flow, causing common age-related disease states such as hypertension, diabetes, and sarcopenia [1, 2]. Since reduced tongue pressure is related to sarcopenia or sarcopenic dysphagia [3], and low-grade inflammation is also reported to be associated with endothelial dysfunction [4] and arterial stiffness (atherosclerosis) [5], an association may exist between tongue pressure and subclinical atherosclerosis by indicating the presence of low-grade inflammation.

On the other hand, platelet levels act as an indicator of endothelial repair (including atherosclerosis and angiogenesis) [6], and sarcopenia is reported to be associated with lower skeletal capillarization [7]. Platelet levels may therefore influence the association between tongue pressure and subclinical atherosclerosis; however, no studies on this have been reported.

In addition, a previous study reported that hypertension plays a central role in muscle capillarization during aging, and other components of metabolic syndrome do not result in additional significant changes in the aged skeletal muscle capillary network [8]. That study indicates that the clinical importance of the influence of tongue pressure on subclinical atherosclerosis is especially true in elderly subjects with hypertension.

Following up on those previous studies, we found and reported that the maintenance of muscle strength of hypertensive elderly men with high, but not those with low, platelet counts can be expected to show a higher rate of progression of atherosclerosis as a form of compensation for age-related disruption of microvascular endothelium [9].

However, evidence for the unfavorable consequences of reduced muscle strength and subclinical atherosclerosis (not as a form of compensation for age-related disruption of microvascular endothelium) was limited at that time.

We therefore conducted a cross-sectional study of 342 hypertensive elderly Japanese men aged 60–89 who participated in an annual health check-up in 2015 and 2016.

## Methods

### Study populations

The original population included 698 men 60 to 89 years old residing in rural communities in Goto city in western Japan who undertook a general medical check-up in 2015 and 2016 as recommended by the Japanese government.

Normotensive subjects (*n* = 315) and subjects with a history of stroke (*n* = 27) were excluded from the study population. To avoid the influence of chronic disease and malnutrition, subjects with low body mass index (BMI) < 18.5 kg/m^2^ (*n* = 8) were also excluded. Additionally, subjects without platelet data (*n* = 3) and tongue pressure data (*n* = 3) were also excluded. The remaining participants, comprising 342 hypertensive subjects at a mean age of 72.7 years (standard deviation (SD) 7.5, range 60–89) were enrolled in the study.

### Data collection and laboratory measurements

Trained interviewers obtained information on clinical characteristics. Body weight and height were measured with an automatic body composition analyzer (BF-220; Tanita, Tokyo, Japan) and BMI (kg/m^2^). Systolic and diastolic blood pressures were recorded at rest.

Fasting blood samples were collected in a EDTA-2K tube, and a siliconized tube. Serum triglycerides (TG) and creatinine were measured enzymatically. HDL-cholesterol (HDL) was measured using a direct method, and hemoglobin A1c (HbA1c) was measured using the latex coagulation method at SRL, Inc. (Tokyo, Japan). Estimated glomerular filtration rate (eGFR) was estimated by using an established method recently proposed by a working group of the Japanese Chronic Kidney Disease Initiative [10]. According to this adaptation, eGFR (mL/min/1.73 m^2^) = 194 × (serum creatinine (enzyme method))^−1.094^ × (age)^−0.287^. Tongue pressure was evaluated by the method proposed by Tsuga et al. using the JMS tongue pressure measurement device, Orarize (TPM-01, JMS Co., Ltd. Hiroshima, Japan) [11].

Measurement of carotid intima-media thickness (CIMT) was determined by ultrasonography of the left and right common carotid arteries by an experienced vascular technician using a LOGIQ Book XP with a 10-MHz transducer (GE Healthcare, Milwaukee, WI, USA). Maximum values for the left and right CIMT were calculated using automated digital edge-detection software (Intimascope; MediaCross, Tokyo, Japan) and a protocol that has been described in detail elsewhere [12]. The values of right and left CIMT without plaque measurement were calculated, and the max CIMT value was used for analysis. The reproducibility of CIMT measurements by max values of Intimascope for our part of the study population (*n* = 30) was shown to be satisfactory: the respective intra-observer variations for CIMT assessed by two examiners were simple correlation coefficients (*r*) = 0.94 (*p* < 0.001) and *r* = 0.83 (*p* < 0.001), and the inter-observer variation was *r* = 0.86 (*p* < 0.001). Since a previous study reported normal CIMT value as < 1.1 mm, we defined subclinical carotid atherosclerosis as a CIMT ≥ 1.1 mm [13]. Hypertension was defined as a systolic blood pressure ≥ 140 mmHg and/or a diastolic blood pressure ≥ 90 mmHg.

### Statistical analysis

Since platelet levels serve as indicators of vascular repair activity [6, 14–17], subjects were stratified by platelet level (median value) as in a previous study [9].

Characteristics of the study population by platelet level were expressed as mean ± standard deviation, as were platelet level-specific characteristics of the study population based on tongue pressure. To evaluate the influence of age on platelet count and platelet level-specific influence of age on CIMT, simple correlation coefficients (*r*) were calculated. For total and platelet level-specific subjects, odds ratios (ORs) and 95% confidence intervals (CIs) for subclinical carotid atherosclerosis (CIMT ≥ 1.1 mm) associated with tongue pressure were calculated with the aid of logistic regression models.

Our study population was compromised by participants with hypertension, and a previous study reported that systolic but not diastolic hypertension is associated with an increase in carotid atherosclerosis [18]. Since our present study dealt with CIMT, instead of using diastolic, systolic blood pressure should be taken as a confounding factor.

Moreover, the aim of our study was to investigate the influence of platelet levels on the association between muscle strength and subclinical atherosclerosis, so that, in this regard, platelet level could indicate platelet activity. However, evidence for the association between platelet level and platelet activity is limited. Therefore, its influence on platelet activity, which is associated with both endothelial repair and muscle strength, should be taken as a confounding factor in our study.

Since alcohol consumption and smoking are reportedly associated with platelet function [19–21] and those factors are also known as risk factors for muscle strength loss [22, 23], both alcohol intake and smoking status should also be considered confounding factors in our study.

Moreover, platelets perform an important role in endothelial repair in conjunction with bone marrow-derived endothelial progenitor cells such as CD34-positive cells [14–16, 24], and active endothelial repair leads to a decrease in the endothelial cell count (consumptive reduction) [25]. Since the circulating CD34-positive cell count is reported to be associated with muscle strength in hypertensive men [26] as well as with platelet count [25, 27], factors that exert a strong influence on circulating CD34-positive cell levels, such as antihypertensive medication use [28], BMI, and HbA1C [25], also should be considered confounding factors for our analyses.

Furthermore, triglycerides and HDL-cholesterol, which are known indicators of sarcopenia risk [29], should also be taken as confounding factors in our analyses because circulating CD34-positive cell levels have been shown to be a determinant factor for the association between the two previously mentioned factors and blood pressure level [30, 31], while platelets and circulating CD34-positive cells act as indicators of the activity of the vicious cycle of hypertension and endothelial dysfunction [25]. Finally, renal function, which can be evaluated in terms of eGFR, is also reportedly associated with platelet levels [32] and atherosclerosis [33]. For these reasons, we think that, in addition to age, all the factors discussed above are important and thus could have a confounding effect on the results presented here.

Therefore, three different approaches were used for making adjustments for confounding factors. First, we made a non-adjusted model. Second, we adjusted only for age. Third, we included other potential confounding factors, namely systolic blood pressure (mmHg), antihypertensive medication use (yes, no), BMI (kg/m^2^), smoking status (never-smoker, former smoker, current smoker), alcohol consumption [never-drinker, former drinker, current drinker (< 23 g/week, 23 g/week, ≤ 46 g/week, 46 g/week, ≤69 g/week, < 69 g/week)], TG (mg/dL), HDL-cholesterol (mg/dL), antilipidemic medication use (yes, no), HbA1c (%), and eGFR (ml/min/1.73 m^2^). We also used continuous variables for age, systolic blood pressure, BMI, TG, HDL-cholesterol, HbA1c, and eGFR, as well as categorized variables for smoking status, alcohol consumption, antihypertensive medication use, and antilipidemic medication use.

All statistical analyses were performed with the SAS system for Windows (version 9.4; SAS Inc., Cary, NC). Values of *p* < 0.05 were regarded as being statistically significant.

## Results

For all study subjects, age was found to be slightly but significantly inversely associated with platelet count: *r* = -0.12 (*p* = 0.022). The median value for platelets among the study population was 21.4 × 10^4^/μL. A total of 99 subjects were defined as having subclinical carotid atherosclerosis.

### Characteristics of the study population based on platelet levels

**Fig. 1:**
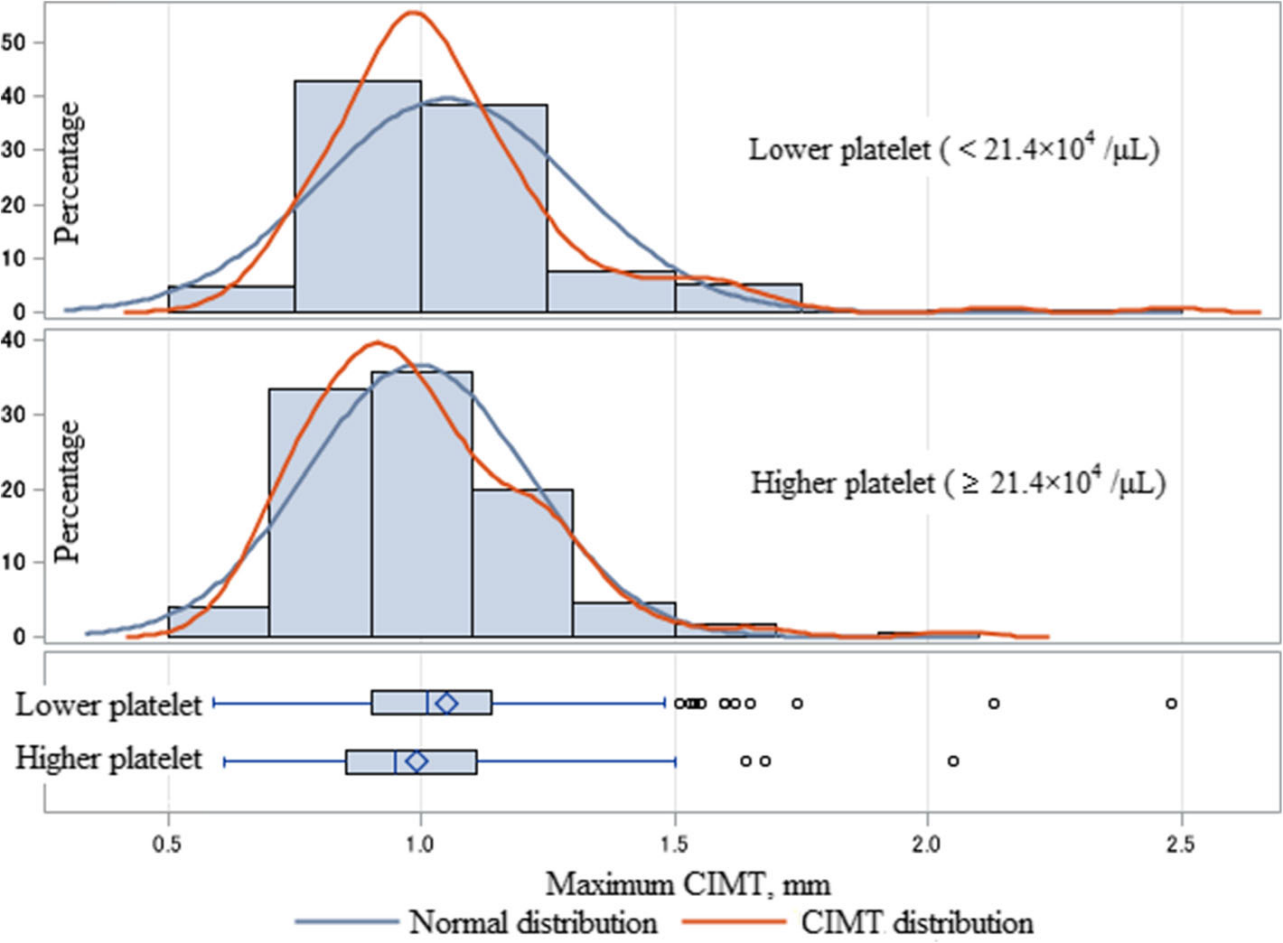
Distribution of maximum carotid intima-media thickness (CIMT) by platelet levels

Table 1 (Fig. 1) shows the characteristics of the study population by platelet level. Subjects with low platelet levels showed significantly higher values for age and CIMT, but significantly lower values for diastolic blood pressure and TG than subjects with high platelets levels.

**Table 1:** Platelet level-specific characteristics of the study population

	Lower platelet count (< 21.4 × 10^4^/μL)	Higher platelet count (≥ 21.4 × 10^4^/μL)	*p*
No. at risk	171	171	
Age, years	74.2 ± 7.6	71.1 ± 7.1	< 0.001
Systolic blood pressure, mmHg	154 ± 15	154 ± 14	0.729
Diastolic blood pressure, mmHg	86 ± 12	90 ± 10	0.003
Antihypertensive medication use, %	57.9	50.9	0.194
Body mass index, kg/m^2^	24.3 ± 2.9	23.9 ± 2.9	0.208
Current drinker, %	52.6	53.2	0.914
Current smoker, %	15.8	18.7	0.476
Serum triglycerides (TG), mg/dL	101 ± 52	117 ± 71	0.018
Serum HDL-cholesterol (HDL), mg/dL	54 ± 14	57 ± 14	0.111
Antilipidemic medication use, %	16.4	20.5	0.333
Serum HbA1c, %	5.8 ± 0.9	5.9 ± 0.6	0.599
Serum creatinine, mg/dL	0.88 ± 0.18	0.92 ± 0.58	0.452
Estimated glomerular filtration rate (eGFR), mL/min/1.73m^2^	67.8 ± 15.4	70.1 ± 16.4	0.179
Maximum carotid intima-media thickness (CIMT), mm	1.05 ± 0.25	0.99 ± 0.22	0.021
Platelets, × 10^4^/μL	17.4 ± 2.8	25.7 ± 4.6	< 0.001

Values: mean ± standard deviation

For subjects with low platelet levels, even CIMT shows no significant association with age (*r* = 0.11, *p* = 0.139), while a significantly positive association was observed for subjects with high platelet levels (*r* = 021, *p* = 0.006).

Characteristics of the study population based on tongue pressure levels and stratified by platelet levels are shown in Table 2. For subjects with both low and high platelets, tongue pressure levels are significantly inversely associated with age, while for subjects with high platelets, tongue pressure shows a significant positive association with current smoker status.

**Table 2:** Platelet level-specific characteristics of the study population based on tongue pressure level

	Tongue pressure tertiles			*p*
	T1 (low)	T2	T3 (high)	
Lower platelet count				
No. at risk	55	64	52	
Age, years	77.3 ± 6.9	73.7 ± 7.2	71.6 ± 7.7	< 0.001
Systolic blood pressure, mmHg	155 ± 14	153 ± 16	153 ± 14	0.801
Diastolic blood pressure, mmHg	85 ± 11	86 ± 14	87 ± 9	0.718
Antihypertensive medication use, %	50.9	50.0	57.7	0.908
Body mass index, kg/m^2^	22.9 ± 2.3	25.2 ± 2.9	24.7 ± 3.1	< 0.001
Current drinker, %	50.9	50.0	57.7	0.682
Current smoker, %	14.5	14.0	19.2	0.719
Serum triglycerides (TG), mg/dL	98 ± 54	106 ± 54	97 ± 49	0.585
Serum HDL-cholesterol (HDL), mg/dL	56 ± 15	53 ± 13	55 ± 13	0.400
Antilipidemic medication use, %	14.5	17.2	17.3	0.907
Serum HbA1c, %	5.9 ± 1.1	5.7 ± 0.5	5.9 ± 0.6	0.357
Serum creatinine, mg/dL	0.91 ± 0.17	0.89 ± 0.20	0.85 ± 0.17	0.222
Estimated glomerular filtration rate (eGFR), mL/min/1.73m^2^	64.6 ± 13.2	67.4 ± 16.6	71.5 ± 15.6	0.694
Maximum carotid intima-media thickness (CIMT), mm	1.07 ± 0.23	1.08 ± 0.30	1.00 ± 0.19	0.218
Higher platelet count				
No. at risk	58	51	62	
Age, years	74.7 ± 7.2	71.6 ± 7.0	67.5 ± 5.1	< 0.001
Systolic blood pressure, mmHg	154 ± 12	155 ± 15	153 ± 15	0.736
Diastolic blood pressure, mmHg	88 ± 11	89 ± 10	92 ± 9	0.088
Antihypertensive medication use, %	48.3	52.9	51.6	0.881
Body mass index, kg/m^2^	23.8 ± 2.9	23.6 ± 2.5	24.2 ± 3.13	0.490
Current drinker, %	46.6	50.9	61.3	0.255
Current smoker, %	8.6	21.6	25.8	0.045
Serum triglycerides (TG), mg/dL	104 ± 51	119 ± 73	127 ± 83	0.188
Serum HDL-cholesterol (HDL), mg/dL	57 ± 15	56 ± 14	57 ± 14	0.822
Antilipidemic medication use, %	19.0	17.6	24.2	0.655
Serum HbA1c, %	5.8 ± 0.7	6.0 ± 0.7	5.9 ± 0.6	0.657
Serum creatinine, mg/dL	0.96 ± 0.82	0.94 ± 0.54	0.86 ± 0.25	0.633
Estimated glomerular filtration rate (eGFR), mL/min/1.73m^2^	68.8 ± 16.8	68.8 ± 16.8	72.3 ± 15.8	0.400
Maximum carotid intima-media thickness (CIMT), mm	1.03 ± 0.23	0.98 ± 0.22	0.96 ± 0.21	0.217

Values: mean ± standard deviation. Tongue pressure level tertiles: < 28.7 kPa, 28.7–36.4 kPa, and > 36.4 kPa

### Association between tongue pressure and subclinical carotid atherosclerosis in total subjects

ORs and 95% CIs for subclinical carotid atherosclerosis in total subjects are shown in Table 3. A significant inverse association was observed in crude model. After adjustment for age, however, this relationship became insignificant, although an inverse tendency was seen for the association between tongue pressure and subclinical carotid atherosclerosis.

**Table 3:** Odds ratios (ORs) and 95% confidence intervals (CIs) for subclinical carotid atherosclerosis

	Tongue pressure			*p* for trend	1 SD increment in tongue pressure	
	T1 (low)	T2	T3 (high)			
Total subjects						
No. at risk	113	115	114			
No. of cases (percentage)	42 (37.2)	33 (28.7)	24 (21.1)			
Crude ORs	1	0.68 (0.39, 1.19)	0.45 (0.25, 0.81)	0.008	0.78 (0.61, 0.98)	
Age-adjusted ORs	1	0.78 (0.44, 1.38)	0.60 (0.32, 1.13)	0.110	0.88 (0.68, 1.13)	
Multivariable ORs	1	0.71 (0.39, 1.30)	0.57 (0.30, 1.11)	0.095	0.86 (0.65, 1.12)	

Multivariable ORs: adjusted further for age, systolic blood pressure, antihypertensive medication use, body mass index, smoking status, alcohol intake, serum triglycerides, serum HDL-cholesterol, antilipidemic medication use, HbA1c, and eGFR. Tongue pressure level tertiles: < 28.7 kPa, 28.7–36.4 kPa, and > 36.4 kPa. Subclinical carotid atherosclerosis is defined as a carotid intima-media thickness ≥ 1.1 mm. *SD* standard deviation. A 1 SD increment in tongue pressure is 10.4 kPa

### Association between tongue pressure and subclinical carotid atherosclerosis in relation to platelet levels

ORs and 95% CIs of subclinical carotid atherosclerosis in relation to platelet levels are shown in Table 4.

**Table 4:** Odds ratios (ORs) and 95% confidence intervals (CIs) for subclinical carotid atherosclerosis based on platelet level

	Tongue pressure			*p* for trend	1 SD increment in tongue pressure
	T1 (low)	T2	T3 (high)		
Lower platelet count					
No. at risk	55	64	52		
No. of cases (percentage)	24 (43.6)	19 (29.7)	10 (19.2)		
Crude ORs	1	0.55 (0.26, 1.16)	0.31 (0.13, 0.74)	0.007	0.55 (0.38, 0.81)
Age-adjusted ORs	1	0.60 (0.28, 1.29)	0.35 (0.14, 0.87)	0.023	0.59 (0.39, 0.87)
Multivariable ORs	1	0.61 (0.26, 1.44)	0.35 (0.13, 0.91)	0.031	0.54 (0.35, 0.85)
Higher platelet count					
No. at risk	58	51	62		
No. of cases (percentage)	18 (31.0)	14 (27.5)	14 (22.6)		
Crude ORs	1	0.84 (0.37, 1.93)	0.65 (0.29, 1.46)	0.297	0.99 (0.72, 1.35)
Age-adjusted ORs	1	1.05 (0.44, 2.51)	1.10 (0.44, 2.79)	0.834	1.27 (0.88, 1.83)
Multivariable ORs	1	0.92 (0.37, 2.30)	1.11 (0.42, 2.29)	0.846	1.31 (0.87, 1.96)

Multivariable ORs: adjusted further for age, systolic blood pressure, antihypertensive medication use, body mass index, smoking status, alcohol intake, serum triglycerides, serum HDL-cholesterol, antilipidemic medication use, HbA1c, and eGFR. Tongue pressure level tertiles: < 28.7 kPa, 28.7–36.4 kPa, and > 36.4 kPa. Lower platelet count is defined as < 21.4 × 10^4^/μL. Subclinical carotid atherosclerosis is defined as a carotid intima-media thickness ≥ 1.1 mm. *SD* standard deviation. A 1 SD increment in tongue pressure is 10.4 kPa

For subjects with low platelet levels, a significantly inverse association was detected between tongue pressure and subclinical carotid atherosclerosis. This association remained unchanged even after adjustment for age and other known cardiovascular risk factors.

And even though no such significant association was observed for subjects with high platelet levels, an inverse tendency was observed for the association between tongue pressure and subclinical carotid atherosclerosis. However, after adjustment for age, this tendency became positive, although the statistical power remained non-significant. Even after further adjustments for other known cardiovascular risk factors, this non-significant positive tendency remained unchanged.

### Effects of interaction between tongue pressure and two platelet categories on subclinical carotid atherosclerosis

We also investigated the effects of interaction between tongue pressure and two platelet categories (low and high) on subclinical carotid atherosclerosis. A significant interaction between tongue pressure and platelet category was observed, with crude, age-adjusted, and multivariable *p* values of 0.021, 0.018, and 0.011, respectively, for the effect of this interaction on subclinical carotid atherosclerosis.

## Discussion

The major finding of the present study is that independent of known cardiovascular risk factors, tongue pressure is inversely associated with subclinical carotid atherosclerosis in hypertensive elderly men with low platelet, but not high platelet, levels.

Reduced tongue pressure is related to sarcopenia or sarcopenic dysphagia [3]. Low-grade inflammation is known to be associated with decreased muscle mass as well as the development of functional disability in elderly populations [34–36]. Since atherosclerosis is recognized as a chronic inflammatory disease [37], tongue pressure may be inversely associated with atherosclerosis by indicating the presence of low-grade inflammation. In fact, a previous study of 208 elderly subjects reported that sarcopenia, characterized by a reduction in muscle mass and strength, is associated with subclinical coronary atherosclerosis and endothelial dysfunction [38].

However, a paradoxical association between muscle strength and subclinical atherosclerosis was also revealed in a previous study. Handgrip strength is an efficient tool to evaluate the loss of skeletal muscle mass and function due to its use as a predictor of old age disability [39]. And our previous study with 795 elderly hypertensive Japanese subjects reported that no significant association between handgrip strength and subclinical carotid atherosclerosis was observed for subjects with lower platelet counts, while a significant positive association was observed for subjects with higher platelets [9].

**Fig. 2:**
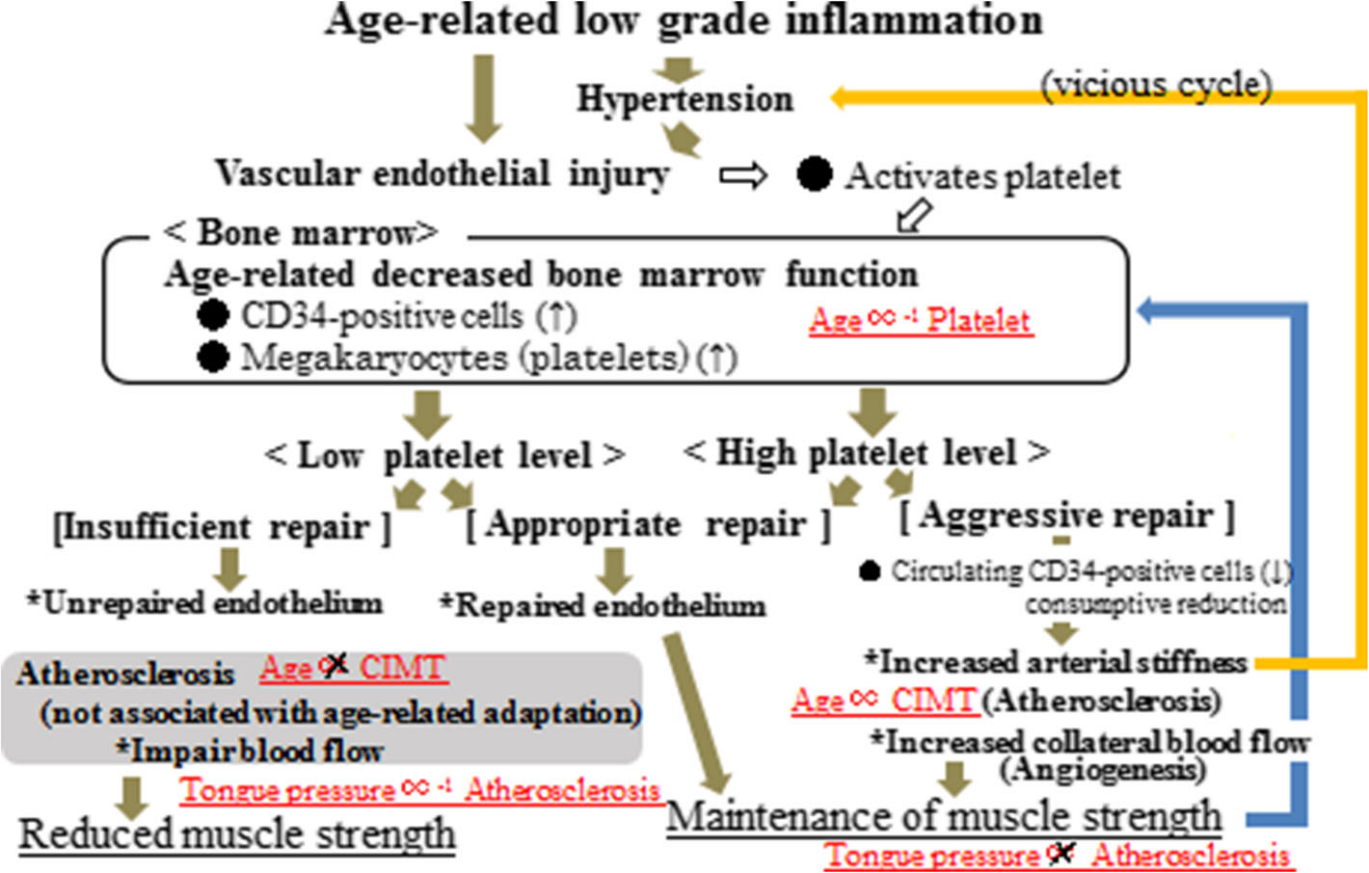
Possible mechanism underlying the association between tongue pressure and atherosclerosis. Relations in red were observed in the present analysis. CIMT: carotid intima-media thickness

Our present study of hypertensive elderly men offers further evidence that tongue pressure is inversely associated with subclinical carotid atherosclerosis only in subjects with low platelet counts. Although the mechanism underlying the findings presented here has not yet been clarified, a possible mechanism to account for these findings is shown in Fig. 2.

Sarcopenia is also associated with the impairment of capillary function [40]. And microvascular endothelium disruption and impaired blood flow could be exacerbated by common age-related diseases, including hypertension and sarcopenia [1]. Since hypertension and atherosclerosis participate in a vicious cycle [25], such age-related diseases may also influence the progression of atherosclerosis in hypertensive subjects.

Furthermore, since endothelial dysfunction has been recognized as one of the initial mechanisms leading to increased arterial stiffness (atherosclerosis) [41], tongue pressure may be inversely associated with subclinical carotid atherosclerosis. However, in our present study, a significant inverse association was observed only in hypertensive subjects with low platelet counts.

Recently, platelets have been reported to play an important role in endothelial repair [14–17]. Platelets have also been revealed to have a significant role in angiogenesis [42]. Therefore, compared to low platelets, high platelets should have a beneficial influence on maintaining blood flow and tongue pressure. However, platelets have also been revealed to play a primary role in inflammation and are also reported to be important for the development of atherosclerotic lesions as an initial actor [43]. Therefore, in subjects with high platelet counts, the existence of atherosclerosis may indicate active endothelial repair and angiogenesis that are crucially important for maintaining tongue pressure. In this connection, a previous study of ours found a significantly positive association between handgrip strength and subclinical atherosclerosis for hypertensive elderly subjects with higher platelet levels [9]. If atherosclerosis is present in subjects with low platelet counts, it can be expected to be characterized by insufficient endothelial repair associated with insufficient angiogenesis, which indicates a risk of reduced tongue pressure. In our present study, no significant association between age and CIMT was observed in subjects with low platelets, while for subjects with high platelets, a significant positive association was observed for these two factors. These results also support the abovementioned mechanism since these results could indicate the presence of active vascular remodeling with age-related changes in subjects with high platelets but not in those with low platelets. In addition, platelet levels were found to be significantly inversely associated with age in the total study population. Therefore, an age-related decrease in bone marrow function might cause insufficient adaptation for age-related inflammation.

Furthermore, angiogenesis is essential for the physical adaptation of skeletal muscle to exercise and occurs in response to the mechanical forces of elevated capillary share stress and cell stretch [44, 45], so maintenance of tongue pressure may also stimulate angiogenesis and contribute to the development of atherosclerosis. These associations may act as a strong confounding factor on the association between tongue pressure and subclinical carotid atherosclerosis.

Our findings therefore indicate that age-related progression of atherosclerosis, but not atherosclerosis itself, might have a beneficial effect on adaptation for age-related inflammation, which is associated with sarcopenia because age-related progression of atherosclerosis involves active endothelial repair and angiogenesis. Our results also revealed that platelet levels could become an important candidate for aiding in the evaluation of adaptation for age-related change. These findings may thus constitute an efficient tool for developing preventive strategy for age-related diseases such as sarcopenia.

However, which factor or factors determine the results presented here has not yet been clarified. Previously, the presence of inflammation-related single nucleotide polymorphism (SNPs) (rs3782886) was revealed to be associated with reduced tongue pressure and short stature in elderly subjects [46]. This study also demonstrated that short stature is positively associated with reduced tongue pressure. Short stature has not only been associated with inflammatory disadvantage [47], higher risk of stroke [48], higher prevalence of subclinical carotid atherosclerosis [49], and higher activity of endothelial repair evaluated in terms of higher platelet levels [27], but also with lower capacity of hematopoietic cell activity [50, 51] and lower capability for endothelial maintenance [52]. All those factors may have confounded our findings, so that further investigation is needed.

Potential limitations of this study warrant consideration. Although active vascular remodeling may have significantly influenced the results, no data was available on the evaluation of current endothelial repair activity such as levels of platelet-derived stromal cell-derived factor-1 [14], angiopoietin 2 [53], and circulating CD34-positive cells [14, 15, 17]. Further analyses that include these data will be necessary. Moreover, because the study population was comprised of subjects who were undertaking an annual health-check-up, selection bias may also be present, since the subjects likely have a higher self-interest in their own health condition. Even though platelet levels have been shown to be a determinant factor for the association between tongue pressure and subclinical carotid atherosclerosis, the exact cutoff point for the influence of this factor remains unknown. Nevertheless, our study observed a significant effect of interaction between tongue pressure and platelet categories (low and high) on subclinical carotid atherosclerosis. Further studies including a precise evaluation of the endothelial repair activity will be necessary. Finally, because this was a cross-sectional study, causal relationships could not be established.

## Conclusions

In conclusion, our study of hypertensive elderly men revealed that independent of known cardiovascular risk factors, tongue pressure is significantly inversely associated with subclinical carotid atherosclerosis in subjects with low, but not in those with high platelet levels. This finding may thus constitute an efficient tool for clarifying the background mechanism of age-related diseases.

## Abbreviations

BMI: Body mass index
CIMT: Carotid intima-media thickness
CIs: Confidence intervals
eGFR: Estimated glomerular filtration rate
HbA1c: Hemoglobin A1c
HDL: HDL-cholesterol
ORs: Odds ratios
SD: Standard deviation
TG: Triglycerides
